# Prevalence of child marriage and its impact on fertility outcomes in 34 sub-Saharan African countries

**DOI:** 10.1186/s12914-019-0219-1

**Published:** 2019-12-19

**Authors:** Sanni Yaya, Emmanuel Kolawole Odusina, Ghose Bishwajit

**Affiliations:** 1 School of International Development and Global Studies, University of Ottawa, Ottawa, Canada; 2 The George Institute for Global Health, The University of Oxford, Oxford, UK; 3 Department of Demography and Social Statistics, Federal University, Oye, Ekiti Nigeria

**Keywords:** Women, Child marriage, Legal age, Early fertility, Global health, Sub-Saharan Africa

## Abstract

**Background:**

The issue of child marriage is a form of human rights violation among young women mainly in resource-constrained countries. Over the past decades, child marriage has gained attention as a threat to women’s health and autonomy. This study explores the prevalence of child marriage among women aged 20–24 years in sub-Saharan Africa countries and examines the association between child marriage and fertility outcomes.

**Methods:**

Latest DHS data from 34 sub-Saharan African countries were used in this study. Sixty thousand two hundred and fifteen women aged 20–24 years were included from the surveys conducted 2008–2017. The outcome variables were childbirth within the first year of marriage (early fertility), first preceding birth interval less than 24 months (rapid repeat of childbirth), unintended pregnancy, lifetime pregnancy termination, the use of modern contraceptive methods, lifetime fertility and any childbirth. The main explanatory variable was child marriage (< 18 years) and the associations between child marriage and fertility outcomes were examined from the ever-married subsample to estimate odds ratios (ORs) and 95% CIs using binary logistic regression models.

**Results:**

In the study population, the overall prevalence of women who experience child marriage was 54.0% while results showed large disparities across sub-Saharan African countries ranging from 16.5 to 81.7%. The prominent countries in child marriage were; Niger (81.7%), Chad (77.9%), Guinea (72.8%), Mali (69.0%) and Nigeria (64.0%). Furthermore, women who experience child marriage were 8.00 times as likely to have ≥3 number of children ever born (lifetime fertility), compared to women married at ≥18 years (OR = 8.00; 95%CI: 7.52, 8.46). Women who experience child marriage were 1.13 times as likely to use modern contraceptive methods, compared to adult marriage women (OR = 1.13; 95%CI: 1.09, 1.19). Those who married before the legal age were 1.27 times as likely to have lifetime terminated pregnancy, compared to women married at ≥18 years (OR = 1.27; 95%CI: 1.20, 1.34). Also women married at < 18 years were more likely to experience childbirth, compared to women married later (OR = 5.83; 95%CI: 5.45, 6.24). However, women married at < 18 years had a reduction in early childbirth and a rapid repeat of childbirth respectively.

**Conclusion:**

Implementing policies and programmmes against child marriage would help to prevent adverse outcomes among women in sub-Saharan Africa. Also, social change programmes on child-marriage would help to reduce child marriage, encourage the use of modern contraceptive, which would minimize lifetime terminated pregnancy and also children ever born.

## Background

Child marriage is a global issue that cuts across countries, cultures and religions. The phenomenon has been experienced by a large number of women globally [1]. In spite of the widespread efforts to end child marriage, about one-third of the girls in low- and middle-income countries will most likely be married before age 18 due to attained progress levels which are not sustained in many countries and less than 10% of girls will get married before they attain 15 years of age [2, 3]. In resource-constrained settings, the prevalence of child marriage is alarming. More than 67 million women aged 20–24 years were married as adolescents by 2010, with 20% of them from Africa. The indication was that 14.2 million adolescents, who are less than 18 years had been married off annually; making almost 39,000 young women married on a daily basis [2]. This will increase to about 15.1 million girls per year, beginning from 2021 to 2030 [2], should the current trend be allowed to persist. Child marriage is rooted in communities’ socio-cultural practices and is an act of human rights violation [2, 4, 5]. To attain Sustainable Development Goal 5 in Africa, there is much to be done to reduce the prevalence of child marriage, especially in sub-Saharan Africa.

Child brides are prone to domestic violence and are less likely to participate in family decision making due to immaturity and lower socioeconomic status [6–8]. One of the major problems with child marriage is the pressure to raise children while they are still children themselves and have limited knowledge about sexual and reproductive life. Research evidence indicates that child marriages are associated with many adverse reproductive outcomes such stillbirth, miscarriage, stunting, underweight, unwanted pregnancies, and abortion [9]. Childhood pregnancy put both the mother and her baby at high risk of adverse reproductive outcomes [2, 10].

More so, complications in pregnancy and delivery are prominent determinants of morbidity (obstetric fistula, HIV/AIDS) and mortality among young women in low- and middle-income countries [2, 9, 11]. International agreements to protect the rights of young women in child marriage include the 1989 United Nations Convention on the Rights of the Child (CRC) [4] and the 1990 African Charter on the Rights and Welfare of the Child (ACRWC) [5]. Also the Programme of Action adopted by the International Conference on Population and Development (ICPD) in 1994 has as part of its activities the protection of young women in child marriage [12]. Article 16(2) of the Convention on the Elimination of all Forms of Discrimination Against Women (CEDAW) states that “women should have the same right as men to freely choose a spouse and to enter into marriage only with their free and full consent” and that the “betrothal and marriage of a child shall have no legal effect [2]. In 2010, about 158 countries confirmed that 18 years was the minimum legal age for marriage. However, in 146 countries, state or customary law allows girls younger than 18 to marry with the consent of parents or other authorities; while in 52 countries, girls under age 15 can marry with parental consent [2]. In 2014, almost all African Union member countries signed some of these laws which emphasise that the minimum age for marriage is 18 [13].

Overall, the political will to implement marriage laws varies substantially across sub-Saharan African countries. Whereas about 90% of the countries in sub-Saharan Africa region (37 out of 41 countries) have legislated a minimum marriage age of 18 years for women, however, one-third of them permit marriage below age 18 years with parental consent, hence creating a compromise for parents to marry off their daughters before they attain adult age [14]. Unfortunately, marriage laws in several sub-Saharan Africa countries have provisions that allow children to marry in certain circumstances such as under customary law or if they become pregnant irrespective of their age. The incoherence in the legal proscriptions is challenging because child marriage is a long term practice which is culturally acceptable as a rightful approach to protecting young women from premarital sex and the consequences of unintended pregnancy and sexually transmitted infections [15].

Several factors promote child marriage, including incentives to marry out young women to lessen the economic burden on disadvantaged households [16]. Furthermore, the needs to reinforce social ties and protect daughters from sexual adversity as well as the believe of some parents that they can improve their social status by marrying off their daughters to a well-off family [17, 18], are among the leading factors promoting child marriage. Moreover, women’s educational attainment, wealth status, religious belief, and place of residence are associated with child marriage [19, 20]. Elsewhere, the practice of child marriage was found to be most prevalent among young women who live in disadvantaged households, lack school education, and dwell in rural residence [21]. Emerging evidence also reveals that drivers of child marriage are complex especially if it is viewed from the perception of those impacted. Not all girl child marriages are arranged; many girl brides may be interested in the relationship. Also, low investment on girls’ education, social norms, sexual relations, unplanned pregnancy, incomplete education, poverty and unemployment among girls have been identified as factors promoting child marriage [22–24]. Despite efforts, policies and intervention programmes put in place by many countries in sub-Saharan Africa, child marriage remains an issue of grave concern.

The problems associated with child marriage led to the post-2015 Sustainable Development Goal-3 (SDG-3) targeted to help many countries attain landmark progress towards ensuring healthy lives and promoting the well-being for all at all ages [25]. The SDG-3 is vital because child marriage denies young women the privilege of developing their potentials as productive and healthy individuals [26]. It also entrenches young women in poverty and limits their life choices [2, 27]. In spite of the problem of child marriage and its health, reproductive and social outcomes, the issue has not been explored adequately, and the dearth of literature on it may hinder effective efforts, policies and intervention pragrammes especially in sub-Saharan African countries. Studies have revealed that reproductive health programmes targeting youth may not reach those who are mostly at risk [9]. This stresses the need for more research on women who experience child marriage. This study aims to examine the influence of child marriage on reproductive outcomes using DHS datasets from 34 countries.

## Methods

### Data source

This study utilised pooled data from the latest Demographic and Health Surveys (DHS) conducted between 2008 and 2017 across 34 sub-Saharan Africa countries. Demographic and Health Surveys (DHS) are comparable nationally representative household surveys that have been conducted in more than 85 countries worldwide since 1984. The DHS were initially designed to expand on demographic, fertility and family planning data collected in the World Fertility Surveys and Contraceptive Prevalence Surveys, and continue to provide an important resource for the monitoring of vital statistics and population health indicators in low- and middle-income countries. The DHS collects a wide range of objective and self-reported data with a strong focus on indicators of fertility, reproductive health, maternal and child health, mortality, nutrition, and self-reported health behaviours among adults [28]. In this profile, the study presents an overview of the DHS, along with an introduction to the potential scope for these data in contributing to the micro and macro epidemiology fields [29]. DHS datasets are available for researchers through DHS at http://dhsprogram.com/data/available-datasets.cfm. See Table 1 for details of survey countries.

**Table 1:** Distribution of data among women aged 20–24 years in sub-Saharan Africa countries (*n* = 60,215)

Country	Year of survey	Sample size (n)
Angola	2015/2016	1873
Benin	2011/2012	1896
Burkina Faso	2010	2662
Burundi	2016/2017	1619
Cameroon	2011	2128
Chad	2014/2015	2602
Comoros	2012	598
Congo	2011/2012	1309
Cote d’Ivoire	2011/2012	1202
DR Congo	2013	2582
Ethiopia	2016	1991
Gabon	2012	904
Gambia	2013	1366
Ghana	2014	722
Guinea	2012	1150
Kenya	2014	3482
Lesotho	2014	792
Liberia	2013	1030
Madagascar	2008/2009	2415
Malawi	2015/2016	4058
Mali	2012/2013	1602
Mozambique	2011	1969
Namibia	2013	444
Niger	2012	1765
Nigeria	2013	4342
Rwanda	2014/2015	971
Sao Tome & Principe	2008/2009	336
Senegal	2010/2011	2159
Sierra Leone	2013	1661
Tanzania	2015/2016	1693
Togo	2013/2014	955
Uganda	2016	2838
Zambia	2013/2014	1879
Zimbabwe	2015	1220

### Measurement of variables

#### Outcome v0061riables

1. Early fertility: This was measured by childbirth within the first year of marriage using the question about the age at first birth; women who had the first baby within the first year were classified as early, not having childbirth in the first year of marriage, was classified as not early [9]. The association between early fertility and age at first marriage would be of interest.
2. Rapid repeat of childbirth: This was measured by whether the first preceding birth interval was less or more/equal to 24 months. The preceding birth interval is calculated as the difference in months between the current birth and the previous birth, counting twins as one birth. Birth interval of less than 24 months has implications for the health of the mother and the child. Closely-spaced and higher-order births pose a greater risk of infant and child mortality [30]. Psychosocial, educational, medical, and financial outcomes are some of the consequences of rapid repeat birth [31].
3. Unintended pregnancy: This was measured by whether a woman ever had an unintended pregnancy by asking whether she wanted the child at birth, wanted the child later or had not wanted any more children. Participants who wanted the child later or had not wanted anymore children were classified as having an unintended pregnancy. Unintended pregnancies have serious adverse health and economic consequences for the child and mother because they are not wanted or wanted later. It is estimated that half of the pregnancies among 15–19 girls in developing countries are not wanted or wanted later [32].
4. Lifetime pregnancy termination: This was assessed by a question in which participants responded yes or no to if a pregnancy had ever resulted in miscarriage, abortion, or stillbirth. Consequent upon the fact that frequent deaths occur among adolescent mothers due to complications during pregnancies and childbirths [33, 34]. About 3.9 million girls between the ages of 15 to 19 engaged in unsafe abortion every year [32]. Also, evidence revealed the relationship between child marriage and stunting, underweight, miscarriage, and stillbirth [6, 9].
5. Modern contraception: It was assessed with a question about forms of modern contraception such as hormonal methods, barrier methods, and female sterilisation and so forth to identify if a woman had ever used a modern contraceptive method or not. The use of contraceptives can prevent early pregnancies and adverse reproductive consequences [35]. In developing countries, 23 million girls between the ages 15 to 19 experience unmet needs for modern family planning methods [32]. If these needs are met among the adolescents at risk, it could prevent 2.1 million, 3.2 million and 5600 unplanned births, abortions, and maternal deaths, respectively every year [32]. High fertility is risky not for the health of children and mother, but also affects capital investment, economic growth and is a treat to environmental sustainability.
6. Lifetime fertility: The total number of births during the lifetime was measured. Participants were classed as having high fertility if they had ≥3 childbirths, which was the mean value for the variable. As the population of adolescents increases globally, it is projected that by 2030, there will be an increase in adolescent pregnancies with a large percent of it coming from Africa [36].
7. Any childbirth: This was measured using “total children ever born”; where those who reported at least 1 were categorised as Yes, and 0 was categorised as No. High maternal mortality is associated with higher parities and older and younger ages [30].

#### Explanatory variable

The explanatory variable was the age at first marriage. The variable was classified as child marriage when the respondent was < 18 years at marriage [1] and adult marriage when the woman was ≥18 years. The study considered women aged 20–24 only as evident from studies conducted on child marriage [9]. This enhances and affords comparison with similar studies. This way, women below age 18 will not be considered, and biases due to selective survival and forward displacement of age at first marriage would be minimized [37].. In addition, it minimises the possibility of errors due to recall which may occur as ages get farther away from 24. Married women in this study are referred to as ever-married, legally married or those living with their husbands in a consensual union.

#### Covariates

This section provides a profile of the background characteristics of respondents. The analysis of these background characteristics provides the socio-economic context within which the age at marriage, fertility, and fertility-control issues are examined.

1. Age: 20–24 years; The study considered women aged 20–24 as evident from studies conducted on child marriage. Age group 20–24 is a typical age group for the study of child marriage [9, 37, 38];
2. Religion: Every participant’s religious affiliation was measured by one of the following: Christianity, Islam and other religious groups;
3. Place of residence: This was measured by whether a woman resided in the urban area or rural area;
4. Maternal education: This was measured by a question in which each participant responded to one of the following: no education, primary, secondary and tertiary;
5. Sex of household head: This referred to the gender of the household head measured as Male vs Female;
6. Currently working: This was measured with whether the participant was working (i.e., Yes) or not (i.e., No);
7. Read newspaper/magazine: This was measured by a question in which participants responded yes or no. [39];
8. Listen to the radio: This was assessed by a question in which participants responded yes or no [39];
9. Watching TV: This was measured by a question in which participants responded yes or no [39];
10. Wealth Index: DHS include questions about household characteristics and possessions such as materials used for house roof, walls and floors; source of water such as open well, stream, or piped system; presence of durable possessions such as radio, fan, automobile, electricity, television, refrigerator, cooking fuel, furniture; and other attributes related to economic status. Using this information of various indicators, household wealth can be constructed [25]. In this study, economic status is measured by computing a ‘wealth index’ using principal component analysis. The factor loadings and z-scores were calculated. For each household, the indicator values were multiplied by the loadings and summed to produce the household’s wealth index value. The standardized z-score was used to disentangle the overall assigned scores to poorest, poorer, middle, richer, richest.

### Ethical consideration

This study used publicly available data. The ethical procedures for data collection were the responsibility of the institutions that commissioned, funded, or managed the surveys. All DHS are approved by ICF International and Institutional Review Board to ensure that the protocols comply with the U.S. Department of Health and Human Services regulations for the protection of human subjects. Therefore, this study did not require further ethical approval.

### Analytical procedure

We calculated sampling weights to account for stratification and clustering in the sample design. Individual to standard DHS question was managed and analysed. Tests of multicollinearity were done. The study did not violate the multicollinearity assumption with a tolerance value of not less than 0.10. In addition, the correlation between each of the independent variables is less than 0.7. Distribution of respondents’ marital status by socio-demographic characteristics was examined. This is in line with the evidence from the literature [9, 40]. The associations between child marriage and fertility outcomes were examined from the ever-married subsample to estimate odds ratios (ORs) and 95% CIs using the binary logistic regression models to calculate the unadjusted models and those adjusting for demographic characteristics (age, place of residence, religion, level of education, sex of household head, wealth index, working status, read newspaper, listen to radio, watch TV). A significant level of 5% was used in this study. STATA version 14 (STATACorp, College Station, TX) was used for data analysis.

## Results

This section presents socio-demographic characteristics of the respondents - the sample of women aged 20–24 (Never married women included). The results showed about a quarter (26.6%) and one-fifth (20.5%) of women reported ages 20 and 22 years, respectively. While those who reported each of the other ages (21, 23 and 24) were less than one-fifth of the total respondents. About one-third of the study population lived in rural areas (59.8%), and Christianity was reported as the dominant religion (67.0%) among the sampled countries in sub-Saharan Africa. Women with higher education were only 5.6% in this study, while 25.7% of the women had no formal education, 30.9 had primary education, and 37.7% had secondary education. Women from male head households were 73.7% women from the poorest and poorer household categories constituted the same percentage each (18.6%). Women in the richest category constituted 24.1% of the sampled population. More than half (52.0%) of the women indicated were currently working. Women who read newspaper or magazine were only one-quarter of the total women while more than one-third (63.1%) claimed they listened to the radio. Respondents who indicated they watched television were almost half (46.3%) of the total sampled population. See Table 2 for details.

**Table 2:** Distribution of women’s age at marriage by demographic characteristics (*n* = 89,188)

Variable	Sample size (n)	Percent (%)	Child marriage (< 18 years)	Adult marriage (≥18 years)	Never married
			%(95%CI)		
Age (Years)					
20	23,682	26.6	40.5 (39.9–41.2)	20.2 (19.7–20.8)	39.2 (38.6–39.8)
21	15,736	17.6	33.2 (32.5–34.0)	27.1 (26.4–27.8)	39.7 (38.9–40.4)
22	18,287	20.5	37.9 (37.2–38.6)	31.4 (30.7–32.1)	30.7 (30.0–31.4)
23	16,122	18.1	35.1 (34.3–35.8)	37.7 (36.9–38.4)	27.3 (26.6–28.0)
24	15,361	17.2	36.2 (35.4–36.9)	41.5 (40.7–42.3)	22.3 (21.7–23.0)
Place of residence					
Urban	35,853	40.2	24.6 (24.1–25.0)	29.1 (28.6–29.5)	46.4 (45.9–46.9)
Rural	53,335	59.8	45.3 (44.9–45.7)	31.6 (31.2–31.9)	23.1 (22.8–23.5)
Religion					
Christianity	56,691	67.0	30.5 (30.1–30.9)	31.8 (31.5–32.2)	37.7 (37.3–38.1)
Islam	23,642	27.9	48.6 (48.0–49.2)	27.4 (26.9–28.0)	24.0 (23.4–24.5)
Others	4291	5.1	47.1 (45.6–48.6)	30.7 (29.4–32.1)	22.1 (20.9–23.4)
Level of education					
No education	22,953	25.7	61.7 (61.1–62.4)	26.9 (26.3–27.5)	11.4 (11.0–11.8)
Primary	27,563	30.9	45.4 (44.8–46.0)	34.6 (34.0–35.2)	20.0 (19.6–20.5)
Secondary	33,651	37.7	18.1 (17.7–18.5)	31.1 (30.7–31.7)	50.8 (50.2–51.3)
Higher	5008	5.6	4.1 (3.5–4.7)	20.9 (19.8–22.0)	75.0 (73.8–76.2)
Sex of household head					
Male	65,730	73.7	41.2 (40.9–41.6)	33.2 (32.9–33.6)	25.5 (25.2–25.8)
Female	23,458	26.3	25.0 (24.4–25.5)	23.0 (22.4–23.5)	52.0 (51.4–52.7)
Wealth index					
Poorest	16,575	18.6	52.9 (52.1)	30.1 (29.4–30.8)	17.0 (16.4–17.6)
Poorer	16,580	18.6	47.0 (46.3–47.9)	31.8 (31.1–32.5)	21.1 (20.5–21.7)
Middle	16,494	18.5	39.9 (39.2–40.7)	31.3 (30.6–32.0)	28.8 (28.1–29.5)
Richer	18,070	20.3	31.5 (30.8–32.2)	31.9 (31.2–32.6)	36.6 (35.9–37.3)
Richest	21,469	24.1	19.2 (18.7–19.7)	28.2 (27.6–28.8)	52.6 (51.9–53.3)
Currently working					
Yes	44,773	52.0	39.7 (39.2–40.2)	32.1 (31.6–32.5)	28.2 (27.8–28.7)
No	41,336	48.0	34.6 (34.2–35.1)	28.5 (28.1–29.0)	36.8 (36.4–37.3)
Read newspaper/magazine					
Yes	22,453	25.2	16.7 (16.2–17.2)	28.4 (27.8–28.9)	54.9 (54.3–55.6)
No	66,575	74.8	43.8 (43.4–44.1)	31.3 (31.0–31.7)	24.9 (24.6–25.3)
Listen to radio					
Yes	56,235	63.1	31.4 (31.0–31.8)	31.1 (30.7–31.5)	37.5 (37.1–37.9)
No	32,865	36.9	46.5 (45.9–47.0)	29.7 (29.2–30.2)	23.9 (23.4–24.3)
Watch television					
Yes	41,246	46.3	25.1 (24.7–25.5)	29.5 (29.1–29.9)	45.4 (44.9–45.9)
No	47,814	53.7	47.2 (46.8–47.6)	31.5 (31.0–31.9)	21.3 (21.0–21.7)

An examination of child marriage by the age of women revealed that age 20 had the highest (40.5%) child marriage, followed by age 22 (37.9%), age 24 (36.2%), age 23 (35.1%) and age 21(33.2%). Child marriage was more pronounced among respondents in the rural areas (45.3%) compared to urban areas (24.6%) [41] and also among Muslims (48.6%) and other religious adherents (47.1%) compared to Christians (30.5%). The percentage distribution of child marriage by educational attainment followed a negative pattern - the higher the educational attainment, the lower the percentage of child marriage. The highest incidence of child marriage was among the respondents with no formal education (61.7%), while the lowest was found among respondents with higher education (4.1%). The percentage distribution of child marriage by wealth status followed the same pattern. The higher the wealth status, the lower the percentage of child marriage. Child marriage reported in the poorest household category constituted 52.9% of the sampled population, while those in the richest household category constituted 19.2% [23, 38, 39, 42]. Overall, distribution of child marriage according to the sex of the household heads revealed a higher incidence of child marriage among households headed by male (41.2%) compared to those headed by a female (25.0%). More than one-third of respondents who indicated they were currently working (39.7%) and those who indicated otherwise (34.6%) reported child marriage. Among the women who listen to the radio (63.1%), about 31.4% had child marriage, while those who do not listen to the radio had higher child marriage (46.5%). Only 25.1% of the women who watch TV reported child marriage, while 47.2% of those who do not watch TV experienced child marriage. Overall, media users had a reduction in child marriage, compared to non-users. See Table 2 for details.

**Fig. 1:**
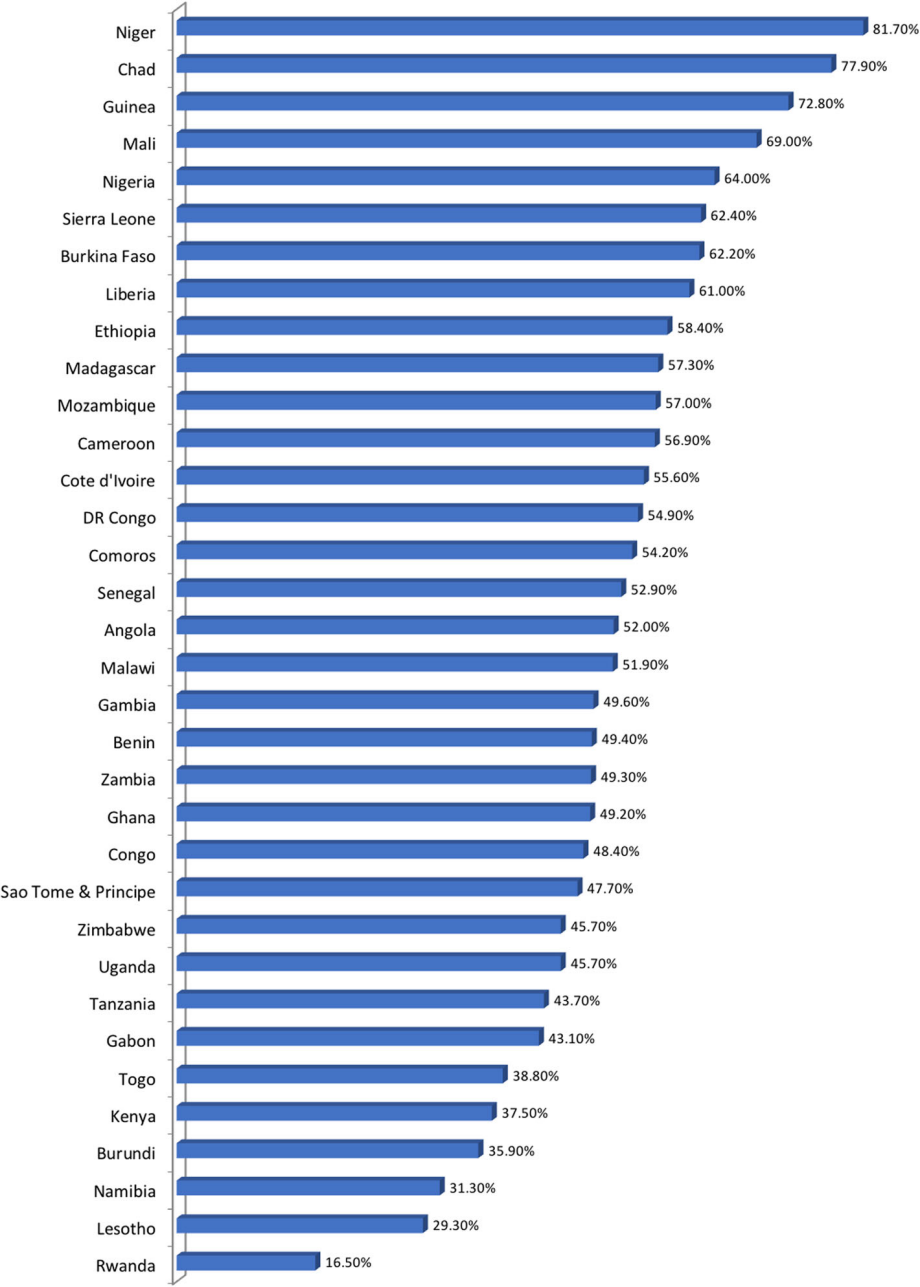
Prevalence of child marriage (< 18 years) across Sub-Saharan Africa countries

Results on the prevalence of child marriage showed large disparities across sub-Saharan African countries between 16.5 to 81.7%. The prominent countries in child marriage were; Niger (81.7%), Chad (77.9%), Guinea (72.8%), Mali (69.0%), Nigeria (64.0%). However, Rwanda reported 16.5%, Lesotho had 29.3%, and Namibia showed 31.3%. Details of women’s age at marriage below 18 years are presented in Fig. 1.

Based on the results, about 56.1% of child marriage had a child in the first year of marriage; for adult marriage, approximately 78.3% gave birth in the first year. More so, about 28.0% of child marriage had childbirth < 24 months of first preceding birth interval. Approximately 34% of child marriage had at least 3 children, compared to 7.2% of adult marriage with the high number of children ever born. Whereas, 20.4% of child marriage had used modern contraceptive methods, compared to about 26% of adult marriage that reported lifetime modern contraceptive methods use. Results showed that 22.3 and 11.6% of child marriage had lifetime unintended and terminated pregnancies, respectively. Further, about 95.6% of child marriage had childbirth. See details in Table 3.

**Table 3:** Distribution of women’s age at marriage by fertility and fertility-control outcomes

Variable	n (%)	Child marriage (< 18 years)	Adult marriage (≥18 years)
		%(95%CI)	
Childbirth in first year of marriage			
Yes	30,024 (64.1)	56.1 (55.5–56.6)	78.3 (77.7–79.0)
No	16,812 (35.9)	43.9 (43.4–44.5)	21.7 (21.0–22.3)
Repeat of childbirth			
< 24 months preceding birth interval	9136 (28.2)	28.0 (27.4–28.5)	29.0 (28.0–30.0)
≥ 24 months preceding birth interval	23,225 (71.8)	72.0 (71.5–72.6)	71.0 (70.0–72.0)
Lifetime fertility			
< 3 children	47,085 (78.2)	66.1 (65.6–66.6)	92.8 (92.5–93.1)
≥ 3 children	13,130 (21.8)	33.9 (33.4–34.4)	7.2 (6.9–7.5)
Modern contraceptive use			
Yes	13,677 (22.7)	20.4 (20.0–20.8)	25.5 (25.0–26.0)
No	46,538 (77.3)	79.6 (79.2–80.0)	74.5 (74.0–75.0)
Unintended pregnancy			
Yes	12,059 (24.3)	22.3 (21.9–22.8)	27.2 (26.6–27.8)
No	37,488 (75.7)	77.7 (77.2–78.1)	72.8 (72.2–73.4)
Terminated pregnancy			
Yes	6509 (11.2)	11.6 (11.3–12.0)	10.6 (10.2–10.9)
No	51,846 (88.8)	88.4 (88.0–88.7)	89.4 (89.1–89.8)
Any childbirth			
Yes	53,316 (88.5)	95.6 (95.4–95.8)	80.0 (79.5–80.5)
No	6899 (11.5)	4.4 (4.2–4.6)	20.0 (19.5–20.5)

The women who experienced child marriage showed 85% reduction in the odds of childbirth in the first year of marriage, compared to those of adult marriage after adjusting for other covariates (OR = 0.15; 95%CI: 0.14, 0.17). Women of child marriage had 14% reduction in childbirth < 24 months of first preceding birth interval, compared to women who married at ≥18 after adjusting for other covariates (OR = 0.25; 95%CI: 0.23, 0.28). Further, women of child marriage were 17.00 times as likely to have ≥3 number of children ever born (lifetime fertility), compared to women who married at ≥18 after controlling for other confounders (OR = 17.00; 95%CI: 15.31, 19.13). Child marriage women were 1.154 times as likely to use modern contraceptive methods, compared to adult married women after controlling for other confounders (OR = 1.54; 95%CI: 1.40, 1.69). Women of child marriage were 1.53 times as likely to have lifetime terminated pregnancy, compared to women who married at ≥18 after adjusting for other covariates (OR = 1.53; 95%CI: 1.33, 1.75). See details in Table 4.

**Table 4:** Association between child marriage, fertility and fertility-control outcomes

Variable	Unadjusted OR	95%CI	Adjusted OR	95%CI
Childbirth in first year of marriage				
Yes	0.35	0.34–0.37*	0.15	0.14–0.17*
No	1.00		1.00	
Repeat of childbirth				
< 24 months preceding birth interval	0.95	0.90–1.01	0.25	0.23–0.28*
≥ 24 months preceding birth interval	1.00		1.00	
Lifetime fertility				
< 3 children	1.00		1.00	
≥ 3 children	6.60	6.27–6.95*	17.11	15.31–19.13*
Modern contraceptive use				
Yes	0.75	0.72–0.78*	1.54	1.40–1.69*
No	1.00		1.00	
Unintended pregnancy				
Yes	0.77	0.74–0.80*	0.70	0.64–0.76
No	1.00		1.00	
Terminated pregnancy				
Yes	1.11	1.06–1.17*	1.53	1.33–1.75*
No	1.00		1.00	

*significant at *p* < 0.05

Adjusted for age, place of residence, religion, education, sex of household head, wealth index, working status, read newspaper/magazine, listen to the radio, watch TV.

## Discussion

This study extensively explored the prevalence of child marriage (marriage at less than 18 years) and its association with fertility and fertility outcomes throughout sub-Saharan Africa countries. The findings revealed an unacceptably high and disproportionate prevalence of child marriage across several sub-Saharan Africa countries [38]; Niger, Chad, Guinea, Mali, Nigeria, Sierra Leone, Burkina Faso, and Liberia were among the leading countries with child marriage. In contrast, each of these countries, Rwanda, Lesotho, and Namibia, had below one-third of their women who were married as children. These findings suggest large disparities in the age at marriage across countries [38], implying that many countries still allowed marriage at very young ages (< 18 years) which could mainly be orchestrated by socio-cultural factors.

Our study showed that about half of sub-Saharan African women aged 20–24 years from rural residence, religious beliefs other than Christianity, less educated, disadvantaged households or those who do not read newspaper or magazine, listen to the radio or watch television were married before the legal age of 18 years and most-at-risk or vulnerable to the practice. These findings are consistent with several reports of previous studies conducted in low- and middle-income countries [37, 41]. It is commonly reported that age at marriage increases with higher socio-economic conditions [43]. This study corroborated findings from other studies that low education, rural residence, low wealth status, disadvantaged households are factors responsible for early child marriage [16–21]. High levels of child marriage persist across several sub-Saharan African countries despite legislative efforts to prevent the practice. It is surprising that though the laws represent a crucial precedence for the protection of human rights and have lasted for decades; our findings suggest that they are inadequate to end the practice. Unfortunately, neither the recent progress in economic and women’s development, nor existing policy or programmatic efforts seems to prevent child marriage in the region [2, 4, 12, 13, 44].

Findings on the association between child marriage, fertility and fertility outcomes showed that women of child marriage had reduced odds in childbirth in the first year of marriage and childbirth less than 24 months first preceding birth interval in comparison to the reference groups. The reduction in the odds of childbirth in the first year of marriage among women of child marriage could be partly attributed to physical and biological immaturity. This may have a great adverse effect on their health and social development. Child marriage has been associated with an increased incidence of poor health [38]. Childbrides are exposed and forced to engage in marital issues, chores, and to take up adult responsibility they have not really prepared to undertake and are not matured enough to undertake. Immaturity of childbrides socially, psychologically, and physiologically may lead to adverse reproductive outcomes. Our understanding is that higher contraceptive prevalence rate leads to lower rates of unintended pregnancy, which is in theory preventive of higher fertility. Nonetheless, it doesn’t account for fertility preference as some women may use modern contraceptive and still desire high parity. In light of these observations, we recommend that policy instruments be developed to correct ‘fertility behaviour’ as an integral part of the strategies to increase the average age of marriage. Previous studies from low- and middle-income countries showed that married adolescent women have higher lifetime fertility, increased use of modern contraceptive methods, more terminated pregnancies, and childbirth than their adult counterparts [41]. High fertility and abortion are inimical to sound sexual and reproductive health of women. Child marriage put women at increased risk of pregnancy complications and maternal mortality. Complications due to childbirths and pregnancies are part of the leading factors in maternal mortality among women aged 15–19 and 20–24 in the world [38, 45, 46]. It has both long and short term consequences most especially in sub- Saharan African countries where there are/ is a high level of poverty, poor/inadequate health facilities (also antenatal and postnatal facilities), low prevalence of contraceptive use, sexual and reproductive education. Negative consequences of child marriage include poor health, low birth weight, premature births and nutritional deficiencies [47].

Increase in the use of modern contraceptive among women married at a young age could be attributed to attainment of the desired number of children at an earlier age, as evident by their high fertility. The results are in line with previous studies that reported an association between child marriage and women’s health or fertility outcomes [9, 10, 26, 41, 48–50]. More so, media use, education, and economic levels were also significant covariates of child marriage infertility outcomes among sub-Saharan Africa women. Mass media can help to educate and address norms, values, social pressure, attitude, and behavior, encouraging the practice of child marriage. Efficient and effective communication can go a long way to reduce the incidence of child marriage.

### Strength and limitation

This study utilised multi-country data coverage with high response rates which can be generalised to other age-groups or country contexts. DHS use standard data collection procedures to ensure reliability, and that survey estimates accurately represent the health situations. In addition, DHS used multistage probabilistic sampling methodology to select clusters and households from geographic-based sampling frames that cover the entire territory of participating countries, a design that translates into naturally occurring population hierarchies. However, this study was based on self-reported data which is subject to recall bias and social desirability [51, 52]; for example, there might be errors in reporting respondents’ age at marriage due to the fact that registration of age system is not documented. In addition, the variable for pregnancy termination did not differentiate between miscarriages and abortion and thus blur the true association that exists between the forms of pregnancy termination and child marriage. Also, the study is limited in its discussion about country differences. Additionally, there was about a nine-year gap in the collection of the data from various countries, and DHS data are cross-sectional, and causality cannot be examined. Finally, we did not consider trichotomizing the exposure variable to < 15, 15–17 and 18 to test for differences by early and very early marital ages. That being said, within policy arena, the focus is usually on zero tolerance on child marriage and not differentiating nor attaching ranking or importance to any form early child marriage, such as labeling early versus very early.

## Conclusion

This study showed that child marriage remains highly prevalent in many sub-Saharan Africa countries. Improved family-planning interventions geared towards married adolescents would help a great deal to tackle the occurrence of child marriage and its outcomes. Prominent factors of child marriage such as poverty and lack of education should be addressed to promote personal development among the girl-child to prevent early marriage and its adverse fertility outcomes. The findings of the study further suggest that health programmes for innovative interventions aimed at discouraging early marriage should be formulated to educate young girls about the negative outcomes of early motherhood.

Furthermore, modern contraceptive methods could help reduce child marriage, especially among women who enter marriage due to unwanted pregnancy. Overall, global consensus points to laws restricting the minimum marriage age at 18 years are essential, and considerable evidence has associated child marriage to adolescent sexual and reproductive health problems. Passing direct and unconditional laws against child marriage to arrest the socio-cultural forces that perpetuate it remains a fundamental approach in curbing the practice in sub-Saharan Africa. Moreover, social change programmes on child marriage, targeting unmarried young women should also be broadened to accommodate interventions for men who are pursing children for marriage.

## Data Availability

Data for this study were sourced from Demographic and Health surveys (DHS) and available here: http://dhsprogram.com/data/available-datasets.cfm.

## Abbreviations

ACRWC: African Charter on the Rights and Welfare of the Child
CEDAW: Convention on the Ellevelimination of all Forms of Discrimination Against Women
CRC: Convention on the Rights of the Child
DHS: Demographic and Health Surveys
ICPD: International Conference on Population and Development
SDGs: Sustainable Development Goals

## References

[1] UNICEF. Progress for children: a world fit for children statistical review. UNICEF; 2007. 72 p.

[2] United Nations Population Fund (2012). Marrying too young: end child marriage [internet].

[3] UNICEF (2012). Progress for children: a report card on adolescents.

[4] Paterson A. United Nations convention on the rights of the child (UNCROC). Fifth Periodic Report by the Government of New Zealand 2015:86.

[5] African Charter on the Rights and Welfare of the Child. In: 2017 [cited 2018 Jun 13].

[6] Santhya KG, Ram U, Acharya R (2010). Associations between early marriage and young Women’s marital and reproductive health outcomes: evidence from India. Int Perspect Sex Reprod Health.

[7] Kidman R (2016). Child marriage and intimate partner violence: a comparative study of 34 countries. Int J Epidemiol.

[8] Delprato M, Akyeampong K, Sabates R, Hernandez-Fernandez J (2015). On the impact of early marriage on schooling outcomes in sub-Saharan Africa and south West Asia. Int J Educ Dev.

[9] Godha D, Hotchkiss DR, Gage AJ (2013). Association between child marriage and reproductive health outcomes service utilization: a multi-country study from South Asia. J Adolesc Health.

[10] Nasrullah M, Muazzam S, Bhutta ZA, Raj A (2014). Girl child marriage and its effect on fertility in Pakistan: findings from Pakistan demographic and health survey, 2006–2007. Matern Child Health J.

[11] Yu SH, Mason J, Crum J, Cappa C, Hotchkiss DR (2016). Differential effects of young maternal age on child growth. Glob Health Action.

[12] International Conference on Population and Development, United Nations, General Assembly, Special Session of the Review and Appraisal of the Implementation of the Programme of Actio7n of the International Conference on Population and Development, United Nations Population Fund, editors. Programme of action: adopted at the International Conference on Population and Development, Cairo, 5–13 September 1994. 2004.

[13] Foley E. The African Committee of Experts on the rights and welfare of the child (ACERWC). An Africa fit for children: 25 years after the adoption of the African Children's charter: accelerating our collective efforts to end child marriage in Africa. Report. 2015:1–15.

[14] Maswikwa, Richter, Kaufman, Nandi (2015). Minimum Marriage Age Laws and the Prevalence Of Child Marriage and Adolescent Birth: Evidence from Sub-Saharan Africa. Int Perspect Sex Reprod Health.

[15] Walker J-A (2012). Early marriage in Africa--trends, harmful effects and interventions. Afr J Reprod Health.

[16] Cader AA. Ending child, early, and forced marriage: SRHR as Central to the Solution. Asian-Pacific Resource & Research Centre for Women (ARROW). 2017:40. ISBN: 978-967-0339-38-2.

[17] Nasrullah M, Zakar R, Zakar MZ, Abbas S, Safdar R, Shaukat M, et al. Knowledge and attitude towards child marriage practice among women married as children-a qualitative study in urban slums of Lahore, Pakistan. BMC Public Health [Internet]. 2014 Dec [cited 2018 Jun 11];14(1). 10.1186/1471-2458-14-1148 PMC4289044 25374265

[18] Nour NM (2009). Child marriage: a silent health and human rights issue. Rev Obstet Gynecol.

[19] Sayi Takudzwa S., Sibanda Amson (2018). Correlates of Child Marriage in Zimbabwe. Journal of Family Issues.

[20] Rumble L, Peterman A, Irdiana N, Triyana M, Minnick E. An empirical exploration of female child marriage determinants in Indonesia. BMC Public Health. 2018;18(1):407. 10.1186/s12889-018-5313-0. 10.1186/s12889-018-5313-0 PMC5869762 29587705

[21] Hotchkiss DR, Godha D, Gage AJ, Cappa C. Risk factors associated with the practice of child marriage among Roma girls in Serbia. BMC Int Health Hum Rights. 2016;16:6. 10.1186/s12914-016-0081-3. 10.1186/s12914-016-0081-3 PMC4736708 26831893

[22] Efevbera Y (2019). Why do young girls marry? A qualitative study on drivers of girl child marriage in Conakry.

[23] Petroni S, Steinhaus M, Fenn NS, Stoebenau K, Gregowski A (2017). New findings on child marriage in sub-Saharan Africa. Ann Glob Health.

[24] Kenny L (2019). Adolescent-led marriage in Somaliland and Puntland: a surprising interaction of agency and social norms. J Adolesc.

[25] Rutstein SO, Staveteig S. Making the Demographic and Health Surveys Wealth Index Comparable. ICF International Measure DHS Calverton, Maryland, USA & United States Agency for International Development (USAID) through the MEASURE DHS project (#GPO-C-08-00008-00). 2012:43.

[26] Oguntade G. Child marriage, an unending abomination in Nigeria | ARFH [Internet]. [cited 2018 Jun 11].

[27] Psaki SR. Addressing early marriage and adolescent pregnancy as a barrier to gender parity and equality in education. United Nations Educational Scientific and Cultural Organization (UNESCO). Education for All Global Monitoring Report. 2015:35. ED/EFA/MRT/2015/PI/23.

[28] Short Fabic M, Choi Y, Bird S (2012). A systematic review of demographic and health surveys: data availability and utilization for research. Bull World Health Organ.

[29] Corsi DJ, Neuman M, Finlay JE, Subramanian S (2012). Demographic and health surveys: a profile. Int J Epidemiol.

[30] Casterline JB. Determinants and consequences of high fertility: a synopsis of the of the evidence – portfolio review. Washington, DC: World Bank.

[31] Boardman LA, Allsworth J, Phipps MG, Lapane KL (2006). Risk Factors for Unintended Versus Intended Rapid Repeat Pregnancies among Adolescents. Journal of Adolescent health.

[32] Darroch J, Woog V, Bankole A, Ashford LS (2016). Adding it up: costs and benefits of meeting the contraceptive needs of adolescents.

[33] WHO. Global Health Estimates 2015: Deaths by cause, age, sex, by country and by region, 2000–2015. Geneva: WHO; 2016.

[34] WHO, UNICEF, UNFPA, World Bank Group and the United Nations Population Division. Trends in maternal mortality: 1990 to 2015: Estimates by WHO, UNICEF, UNFPA, World Bank Group and the United Nations population division. Geneva: WHO; 2015.

[35] WHO. Preventing Early Pregnancy and Poor Reproductive Outcomes among Adolescents in Developing Countries. Geneva: WHO; 2011.

[36] UNFPA (2013). Adolescent pregnancy: a review of the evidence.

[37] Koski A, Clark S, Nandi A (2017). Has child marriage declined in sub-Saharan Africa? An analysis of trends in 31 countries. Popul Dev Rev.

[38] de Groot R, Kuunyem MY, Palermo T (2018). Child marriage and associated outcomes in northern Ghana: a cross-sectional study. BMC Public Health.

[39] Bankole A, Rodríguez G, Westoff CF (1996). Mass media messages and reproductive behaviour in Nigeria. J Biosoc Sci.

[40] Efevbera Y, Bhabha J, Farmer P and Fink G. Girl child marriage, socioeconomic status, and undernutrition: evidence from 35 countries in Sub-Saharan Africa. 10.1186/s12916-019-1279-8 PMC6407221 30845984

[41] Raj A, Saggurti N, Balaiah D, Silverman JG (2009). Prevalence of child marriage and its impact on the fertility and fertility control behaviors of young women in India. Lancet.

[42] Jensen R, Thornton R (2003). Early female marriage in the developing world. Gend Dev.

[43] UNICEF (2014). Ending child marriage: progress and prospects.

[44] Urama K, Ozor N, Acheampong E. Achieving sustainable development goals (SDGs) through transformative governance practices and vertical alignment at the national and subnational levels in Africa. United Nations tools in planning for Sustainable Development 2015:24.

[45] Santhya K, Ram U, Acharya R, Jejeebhoy SJ, Ram F, Singh A (2010). Associations between early marriage and young women's marital and reproductive health outcomes: evidence from India. Int Perspect Sex Reprod Health.

[46] Mokdad AH, Forouzanfar MH, Daoud F, Mokdad AA, El Bcheraoui C, Moradi-Lakeh M, Kyu HH, Barber RM, Wagner J, Cercy K (2016). Global burden of diseases, injuries, and risk factors for young people's health during 1990–2013: a systematic analysis for the global burden of disease study 2013. Lancet.

[47] Raj A, Saggurti N, Winter M, Labonte A, Decker MR, Balaiah D, Silverman JG (2010). The effect of maternal child marriage on morbidity and mortality of children under 5 in India: cross sectional study of a nationally representative sample. BMJ.

[48] Hampton T (2010). Child marriage threatens girls’ health. JAMA..

[49] Kamal SMM (2012). Decline in child marriage and changes in its effect on reproductive outcomes in Bangladesh. J Health Popul Nutr.

[50] Kamal SMM (2011). Socio-economic determinants of age at first marriage of the ethnic tribal women in Bangladesh. Asian Popul Stud.

[51] Yaya S, Oladimeji O, Oladimeji KE, Bishwajit G (2019). Determinants of prenatal care use and HIV testing during pregnancy: a population-based, cross-sectional study of 7080 women of reproductive age in Mozambique. BMC Pregnancy Childbirth.

[52] Yaya S, Buh A, Bishwajit G (2019). Satisfaction with job and family life, and association with smoking and alcohol drinking behaviors among young men in Malawi: analysis from a multiple indicator survey. BMC Res Notes.

